# Course of joint range of motion in children with spinal muscular atrophy receiving disease-modifying treatment

**DOI:** 10.1186/s13023-025-04109-0

**Published:** 2025-11-19

**Authors:** I. L. B. Oude Lansink, J. W. Gorter, W. L. van der Pol, D. R. vd Woude, P. J. Scheiberlich, R. P. A. van Eijk, B. Bartels, A. Beelen

**Affiliations:** 1 Department of Rehabilitation, Physical Therapy Science & Sports, University Medical Center Utrecht, Wilhelmina Children’s Hospital, Utrecht, The Netherlands; 2 Center of Excellence for Rehabilitation Medicine, UMC Utrecht Brain Center, University Medical Center Utrecht, and De Hoogstraat Rehabilitation, Utrecht, The Netherlands; 3 CanChild, Department of Pediatrics, McMaster University, Hamilton, ON Canada; 4 Department of Neurology, UMC Utrecht Brain Center, University Medical Center Utrecht, Utrecht, The Netherlands; 5 Child Development and Exercise Center, Wilhelmina Children’s Hospital, University Medical Center Utrecht, Utrecht, The Netherlands

**Keywords:** Spinal muscular atrophy, Contractures, Joint range of motion, Children, Disease-modifying treatment, Prevention

## Abstract

**Background:**

Progressive decreases in joint range of motion (ROM) is a well-recognized complication in the natural history of spinal muscular atrophy (SMA). How joint ROM evolves in children with SMA receiving disease-modifying treatment (DMT) needs to be documented.

**Purpose:**

To examine the longitudinal course of joint range of motion in young children with SMA receiving disease-modifying therapy.

**Methods:**

We included children with SMA (with 2 or 3 *SMN*2 copies) who started treatment within the first 18 months of life in a prospective national tertiary cohort study. Our examination consisted of joint range of motion of the knee, elbow and wrist; the longitudinal course was studied using linear mixed-effects models.

**Results:**

We analysed 165 visits of 39 children (median age 22 months (interquartile range [6–45])) with treated SMA over a 3-year follow-up period. The median age at start of treatment was 2 months [0–8]. We found an average yearly decline in knee extension mobility of 3°. The overall course of range of motion for elbow and wrist remained stable.

**Conclusion:**

The course of joint mobility in children with SMA, who started treatment with DMT in the first 18 months of life, is characterised by a decline in knee extension and a stable range of motion of wrist and elbow joints. We stress the importance of monitoring knee extension range of motion at least every 6 months and adopting a proactive approach to maintain full knee extension for optimal lifelong mobility.

**Supplementary Information:**

The online version contains supplementary material available at 10.1186/s13023-025-04109-0.

## Introduction

Spinal muscular atrophy (SMA) is an autosomal recessive neuromuscular disease, caused by the homozygous loss of function of the survival motor neuron (*SMN*) *1* gene [1]. It demonstrates a large variation in severity [2], ranging from infantile and childhood to adult onset. This can be explained primarily by the variation in the copy number of the near-homologous second human *SMN* gene (*SMN2*) that produces sufficient amounts of SMN protein for embryonal development, but not for maintenance of motor neuron function. SMA severity correlates inversely with *SMN2* copy number.

For clinical purposes, the SMA classification system recognizes several SMA types in treatment-naïve patients. Type 1 is characterized by infantile onset, stalled gross motor development that impedes the child from learning to sit independently and a median life expectancy of approximately 8 months [3]. Children with SMA type 2 experience onset of symptoms before 18 months of age and achieve the ability to sit. In contrast, children with SMA type 3 experience onset of symptoms after the age of 18 months and achieve the ability to sit and walk independently. In SMA with adult onset, i.e. type 4, limitations may occur in specific higher level functional activities. The natural history of all SMA types is characterized by progression of muscle weakness, resulting in functional loss [4, 5]. 

Pharmacologically increasing intracellular SMN protein concentrations is the aim of the three disease-modifying therapies (DMT) of which the first, Nusinersen (Spinraza^®^), has been approved in the US in December 2016 and in Europe in June 2017. Nusinersen (Spinraza^®^) and Risdiplam^®^ (Evrysdi) increase SMN protein through a splicing modification of the *SMN2* gene, while Onasemnogene abeparvovec (Zolgensma^®^) is an adeno-associated serotype 9 (AAV9)-based gene therapy that restores *SMN*1 in motor neurons. All therapies have been shown to improve survival in SMA type 1 and motor function in SMA type 1–3 [6–9], and presymptomatic treatment yields the best results.

Impaired motor function is not only caused by weakness and fatigability, but also by progressive scoliosis and contractures [10]. Prevalence of contractures in treatment-naive SMA patients varies with age, SMA type and ambulatory status, with a reported prevalence range of 22% to 100% [2, 11–17]. In their 2011 study, Fujak et al. [18] reported that in the upper extremity the most pronounced restriction was observed in the elbow joint, characterized by severe flexion contractures. A characteristic of SMA type II is the early development of restricted radial deviation of the wrist, leading to ulnar deviation contractures in some patients. [18] In the lower extremity, Fujak et al. [12], also reported contractures typically began at the knee, followed by involvement of the hip and ankle.

Prevention of contractures has become an even more important goal of rehabilitative management, as a sufficient joint range of motion is a prerequisite in order to benefit from muscle strength gains as a result of disease-modifying treatment (DMT). However, the course of joint range of motion in treated patients with SMA remains to be documented. We undertook this study to prospectively describe the mean joint range of motion over a 3-year follow-up period, in young children with SMA who started treatment with DMT in the first 18 months of life.

## Method(S)

### Study design

We performed an observational prospective study between April 2021 and August 2024 to evaluate range of motion of three joints (knee, elbow and wrist) in young children with SMA, treated with DMT (who started DMT within the first 18 months of life). We performed assessments during the normal clinic/outpatient visits to minimize the burden for child and parents. Intervals between these visits to the Netherlands SMA Center varied between 4 and 12 months. We shortened the initial 8-monthly interval for assessment of joint range of motion to 4-months to avoid large follow-up gaps due to missed appointments.

Reporting this study conforms to the STROBE (Strengthening the Reporting of Observational Studies in Epidemiology) statement [19]. 

### Setting

We performed this study at the Netherlands SMA Center, at the University Medical Center Utrecht (UMCU), the only hospital in the Netherlands (population 18 million) that provides treatment with DMT for individuals with SMA. Nusinersen first became available in May 2017 [20] for SMA type 1 and from January 2018 for young children (up to the age of 6 years) with SMA types 2 and 3 (compassionate use and early access programs, respectively) and was reimbursed for children up to the age of 9.5 years at the start of treatment as of August 2018.

During the study several changes in the treatment regimen of SMA occurred in the Netherlands:

- Reimbursement of gene therapy (Zolgensma^®^) in December 2021 for presymptomatic cases with 2 or 3 *SMN2* copies.
- The introduction of newborn screening in the Netherlands in June 2022.
- Risdiplam^®^ (Evrysdi) was the third DMT with reimbursement (July 2023) for 2 groups of children: (1) patients with SMA from 2 months to 25 years of age with SMA types 1, 2 or 3, and (2) presymptomatic cases with 1, 2, 3 or 4 *SMN2* copies.

### Participants

All children with a genetically confirmed diagnosis of SMA (a homozygous deletion of the *SMN1* gene) and 2 or 3 *SMN2* copies, who started DMT (Spinraza^®^, Zolgensma^®^ or Risdiplam^®^) within the first 18 months of life were eligible. At the start of the study 30 children with SMA (and 2 or 3 *SMN2* copies) fulfilled the criteria. During the study period children with a new genetically confirmed diagnosis that fulfilled the criteria could potentially be included. We documented age at diagnosis and age at start of DMT.

The Medical Ethics Committee of the University Medical Center Utrecht, The Netherlands classified this study as exempt from the Medical Research Involving Human Subjects Act (20–791); all children and/or parents gave signed informed consent. All participants in this cohort are included in an ongoing prospective population-based cohort study in the Netherlands, approved by the local Medical Ethical Committee (registered at the Dutch registry for clinical studies and trials, NL29692.041.09).

### Assessments of joint range of motion and motor function

Joint range of motion was assessed by four experienced paediatric physiotherapists (DvdW, TR, KS and DvD), who used a universal plastic 18 cm goniometer. Prior to the study, all physiotherapists completed a comprehensive training in standardised goniometry assessment and documentation of range of motion scores according to a standard operating procedure (SOP). The SOP included a detailed description of testing positions and goniometer placement based on standard goniometric measurements (Additional file 1) [21]. To ensure quality and consistency of goniometry assessments throughout the study period, training sessions were repeated every 6 months.

Based on a combination of available historical data [11, 12, 14–16, 18], joint functionality [13, 15], clinical experience, and practical feasibility, we chose to measure the knee, elbow, and wrist joints in this study. For the knee and elbow, we were particularly interested in extension deficits [5, 22–24], as these pose a greater functional problem in SMA, for example in standing and reaching, respectively. Given the emergence of a new phenotype and the uncertainty regarding how different muscle groups might respond to DMT [25], we additionally included other joint motions (elbow flexion, knee flexion in supine/prone, and popliteal angle) to provide a context to contracture development. We collected, the following, quantitative passive ROM (left/right) data: wrist (ulnar/radial deviation), elbow (flexion/extension) and knee (flexion/extension). Goniometry values were recorded to the nearest 5˚.

### Contracture management questionnaire

All participants received rehabilitation care that included an individual plan for contracture management. We documented the type of contracture management using a specifically designed questionnaire (Additional file 2) that covered the use of stretching exercises, orthoses and standing frame in the week before the study visit.

### Statistical analysis

We used descriptive statistics for baseline characteristics; continuous variables (age and duration of DMT) are presented as median (interquartile range (IQR)) given the skewed distribution, and categorical variables (type of DMT) as frequency (percentage). We used linear mixed-effects models (LMMs) to analyse changes in joint range of motion over time where age at assessment was included as the time variable. LMMs account for differences in timing between assessments. The fixed part of the model contained a term for age and age squared to visualize a potential non-linear trajectory; due to small between-subject variability in rates of change over age, only a random intercept per individual was added as a random effect. The average trajectory over age was summarized by a model that only contained a fixed effect for age. The results of the non-linear models are graphically presented with regression lines and its 95% confidence intervals (CI). The results of the linear models are presented in Table 2; p-values were based on a likelihood ratio test with one degree of freedom. We described contracture management by reporting the number of participants for each type of management per age category.

We used R (v4.3.1; R Core Team 2023) for all statistical analyses [26]. LMMs were fitted using *nlme* (v3.1-166) [27] and *ggplot2* (v3.5.1) [28] was used for data visualization.

## Results

### Participants

Forty-nine children with SMA in our national population-based SMA registry were eligible. Seven children and their parents declined to participate. We recruited a total of 42 children (21 male; 21 female) between April 2021 and August 2024. We could not assess three children at baseline as they were restless at the time of assessment; their data were not used in this study. The remaining 39 children were aged between 0 and 6 years at baseline (median: 22 months). The median duration of being treated with DMT at baseline was 14 months (IQR 5–33). All children remained on DMT throughout the study. The demographic and clinical characteristics are summarized in Table 1.

**Table 1:** Demographics and clinical characteristics

Parameters	All participants (*n* = 39)	2 SMN2 copies (*n* = 18)	3 SMN2 copies (*n* = 21)
M: F	20:19	9:9	11:10
Type of DMT, n (% of total)			
- Spinraza^®^	17 (44)	10 (55)	7 (33)
- Zolgensma^®^	21 (54)	7 (39)	14 (67)
- Risdiplam^®^	1 (2)	1 (6)	0 (0)
Age at diagnosis, months*	2 (0–6)	2 (0–3)	2 (0–11)
Age at start of DMT, months	2 (0–8)	2 (0–3)	6 (1–11)
Age at study entry, months	22 (6–45)	31 (9–48)	19 (6–32)
Duration of DMT at study entry, months	14 (5–33)	24 (6–45)	9 (5–25)

DMT: disease-modifying treatment; n: number of participants

All data are described as median (range Q1-Q3), unless otherwise specified

A subset of participants (*n* = 8) altered their type of DMT during the study period. For Table 1, we report the DMT that was most recently administered at the time of study entry

*n = 2 unknown participants’ age of diagnosis

### Longitudinal course of joint range of motion in relation to age

**Fig. 1:**
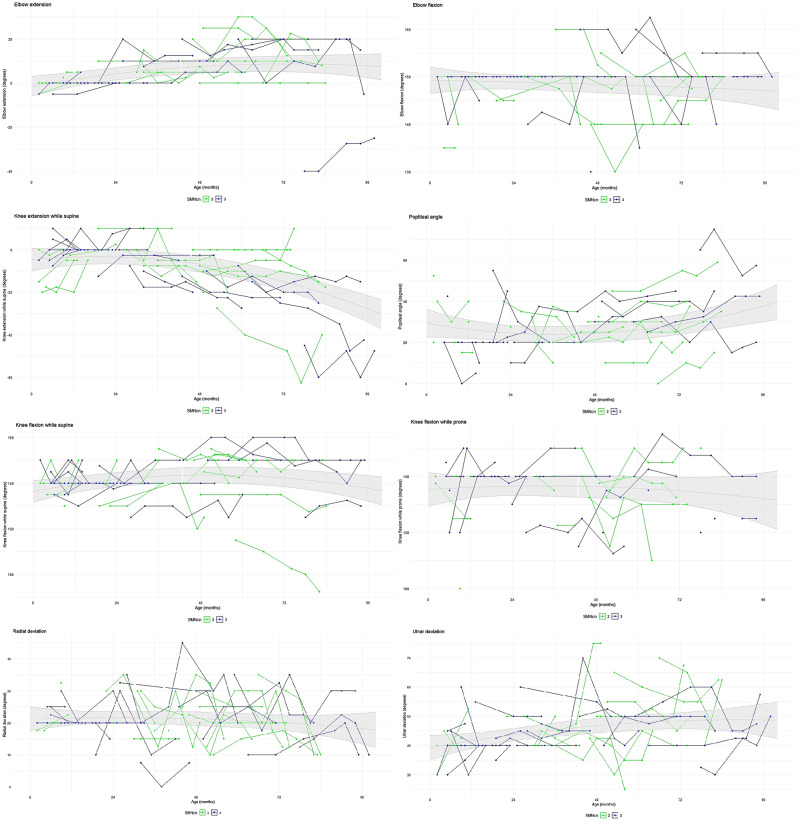
Joint range of motion

We obtained joint range of motion data from a total of 165 visits of 39 children. The average number of visits per child was 4 (min-max 1–7). Thirty-nine children completed their first visit; 38 completed two visits; 33 three visits; 23 four visits; 19 five visits; 9 six visits and 4 seven visits. Median follow-up was 16 months (IQR 8–24). For the LMM analysis we used the average of the measurements of the left and right side, with the assumption that SMA has a nearly symmetrical loss of range of motion [12, 18]. In Fig. 1 we present range of motion data, combined with the non-linear regression lines and its 95% CI. The 95% CIs for the model-estimated baseline and slope parameters are shown in Table 2. Range of motion data for subgroups with either 2 or 3 *SMN2* copies are presented in Additional file 3.

**Table 2:** Model parameters joint range of motion. Intercept and average monthly change in degrees (= slope)

Joint range of motion (ROM)	*N* (*p*)	*N* (ass)	Intercept (SE)	Monthly change ROM (SE)	95% CI of monthly ROM change	*P*-value
EE	39	164	1.507 (1.89)	0.092 (0.034)	0.03; 0.16	0.008
EF	39	155	149.42 (1.05)	-0.022 (0.021)	-0.06; 0.02	0.297
KE	39	165	1.576 (2.24)	-0.246 (0.041)	-0.33; -0.17	< 0.001
POP	39	141	22.727 (2.51)	0.103 (0.049)	0.01; 0.20	0.038
KF-s	39	158	140.78 (1.96)	0.008 (0.035)	-0.06; 0.08	0.824
KF-p	35	107	136.73 (2.29)	-0.026 (0.050)	-0.12; 0.07	0.605
RD	39	156	22.21 (1.35)	-0.030 (0.028)	-0.08; 0.02	0.279
UD	39	156	40.89 (1.56)	0.103 (0.032)	0.04; 0.17	0.002

Model parameter estimates, standard errors, and confidence intervals for the linear mixed-effects models are shown. ROM = joint range of motion; EE = Elbow Extension; EF = Elbow Flexion; KE = Knee Extension; POP = popliteal angle; KF-s = Knee Flexion - supine; KF-p = Knee Flexion - prone; RD = Radial Deviation; UD = Ulnar Deviation; N (p) = number of participants; N (ass) = number of assessments (left = right); SE = standard error; CI = confidence interval

### Knee flexion and extension

We obtained a total of 165 assessments for knee extension, 141 for the popliteal angle, 158 for knee flexion in supine position and 107 for knee flexion in prone position. Our non-linear analysis is shown in Fig. 1.

For knee extension, we found a significant decrease in range of motion over time, a yearly loss of 3° (Table 2).

Additionally, our linear data showed an average increase in range of motion of 1.24°/year (95% CI: 0.12; 2.40) for the popliteal angle and 0.10°/year (95% CI: -0.72; 0.96) for the knee flexion-supine and − 0.31°/year (95% CI: -1.44; 0.84) for the knee flexion in prone position.

In more detail, visual inspection of plotted data showed 8 children, aged between 0 and 2 years, who started with a limited knee extension mobility (< 0°) to improve range of motion in the subsequent 2 years.

### Elbow flexion and extension

We obtained a total of 164 assessments for elbow extension and 155 assessments for elbow flexion. The non-linear model showed a relatively stable range of motion (Fig. 1). We found a yearly change for the elbow extension of 1.10° (95% CI: 0.36; 1.92) and − 0.26° per year (95% CI: -0.72; 0.24) for the elbow flexion.

### Wrist ulnar and radial deviation

We obtained 156 assessments for longitudinal analysis of both ulnar and radial deviation. Our non-linear model is shown in Fig. 1. Our linear analysis showed a decrease in range of motion of -0.36°/year (95% CI: -0.96; 0.24) for radial deviation and an increased range of motion of 0.12°/year (95% CI: 0.48; 2.04) for ulnar deviation.

### Received contracture management

**Fig. 2:**
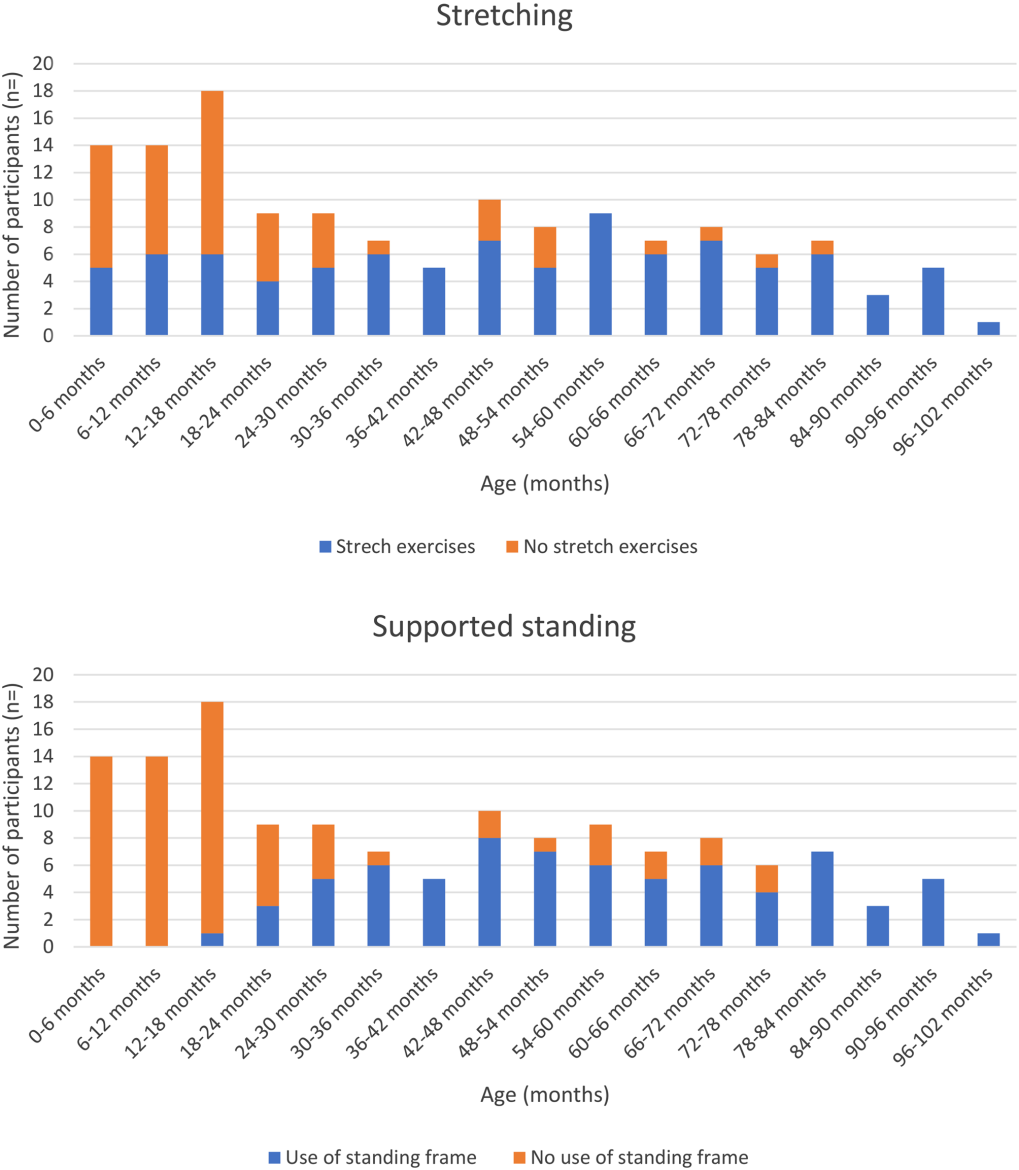

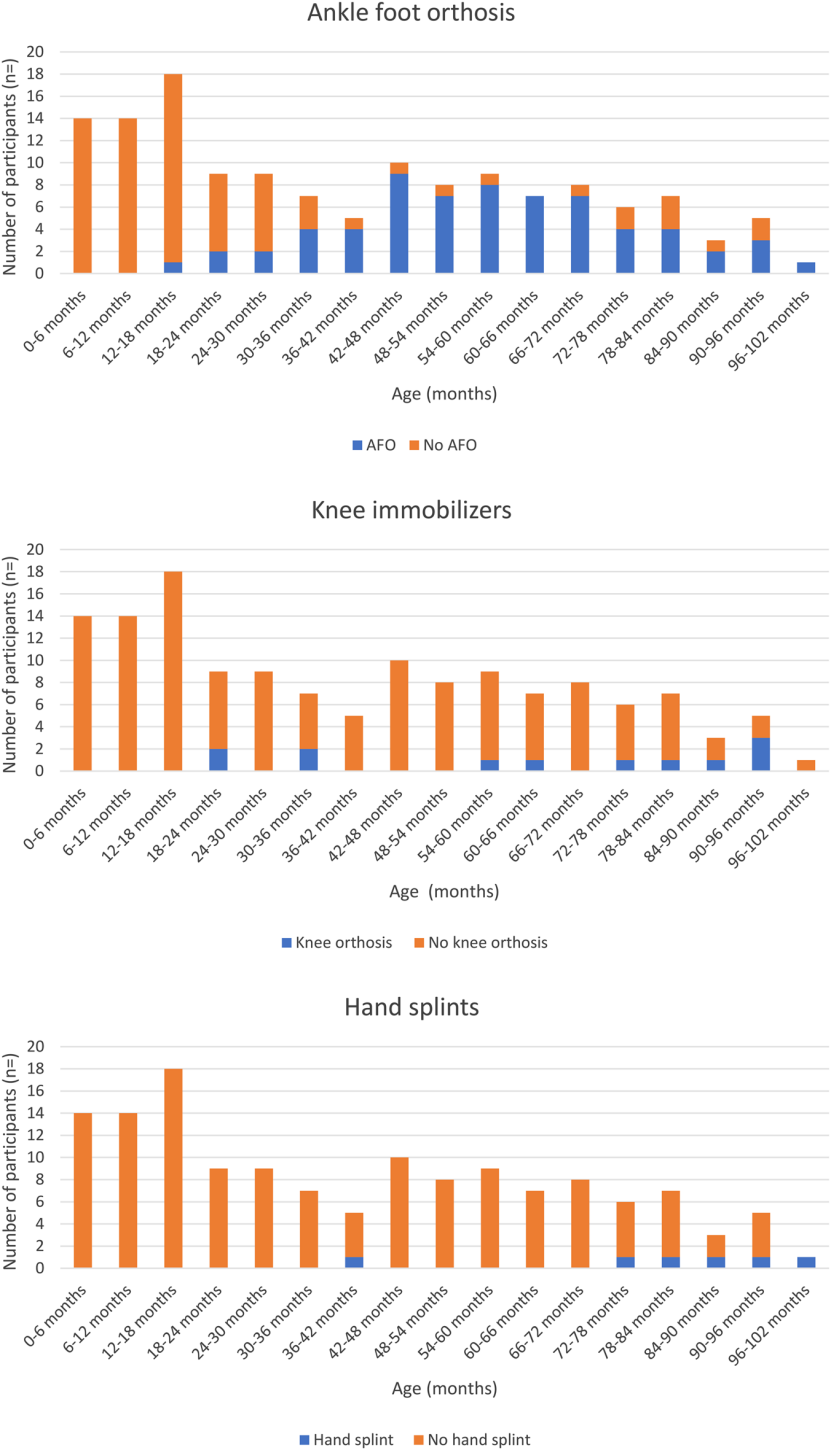
Information on patient reported contracture management, divided in age groups. Reporting on the use of orthoses (knee immobilizers, ankle foot orthoses and hand splints), stretch exercises and supported standing

We obtained a total of 140 questionnaires from 33 children regarding the contracture management regime they received during the period of study participation. The median number of completed questionnaires per child was 4 (IQR 1–7). Figure 2 illustrates the reported use of different interventions for contracture prevention and/or treatment. Subsequently, we show the number of children that use bracing (hand splints/knee immobilizers/AFOs), perform stretch exercises or supported standing. For this population the use of knee immobilizers and hand splints is rare. AFOs are used in a larger group of children, starting from the age of 12–18 months. Most of the children performed stretching exercises from the age of 24–30 months. The standing frame was used throughout the study by 12 children, 4 children started during the study and 17 did not use a standing frame during the study (*n* = 6 children data not available). At 24–30 months 5/9 children used a standing frame; at 78–84 months 7/7 reported the use of a standing frame.

## Discussion

In this prospective study, we evaluated the course of joint range of motion in young children (0–8 years of age) with SMA who received treatment with DMT (Spinraza^®^, Risdiplam^®^ or Zolgensma^®^). The overall range of motion of wrist and elbow remained stable over the age range, but knee extension mobility decreased with an estimated yearly decline of three degrees.

The development of significant joint contractures in treatment-naive children with SMA is common [12, 14, 18]. Despite the fact that others have pointed out the significance of studies on joint range of motion in treated SMA patients [29], the current literature is limited to a case-report [30] and a pilot study [31] with a limited follow-up duration and number of joints studied.

Within the investigated age range, our findings are in line with previous observations regarding joint range of motion patterns of elbow flexion, popliteal angle, or knee flexion (both supine and prone positions) and in particular that the ROM remained stable. Limitations in elbow/knee extension and wrist deviation are well-known complications in untreated children with SMA and we therefore hypothesized that DMT [32–34] would postpone or even prevent contracture development in elbow/knee extension and wrist deviation. Although there are limitations when directly comparing our findings to data from a retrospective cohort study [12] due to clear differences between our longitudinal analysis and the analysis used in the previous studies (i.e. a cross-sectional description per age group), our data may indicate that the pattern of contracture development and progression under DMT may differ from that previously reported. For example, Fujak et al. [18]. reported significant contractures of the elbow, i.e. a 16.7% loss of ROM present in children aged 0–2 years old and 36.1% loss of ROM present in children aged 3–5 years old, while we did not observe limitations in elbow extension. Although we cannot completely exclude the possibility that the observed differences between studies are due to the cross-sectional nature of previous efforts [18]. ,The observation regarding wrist deviation contractures is similar/identical [18].

How do our data compare to what is known from normal development? The values in our group correspond reasonably well with the reference data for elbow extension from children aged 3 to 9 years, showing a mean ROM of 7 degrees [35]. The knee extension observed in children with SMA treated with DMT shows considerably lower joint range of motion compared to the reference norm of 4 degrees reported for both boys and girls, suggesting that in this age range children with SMA deviate from their typically developing peers. To the best of our knowledge, there are no published reference values for wrist deviation in young, normally developing children.

Knee flexion contractures are the most common contractures in treatment-naïve children with SMA type 2 [12]. This probably reflects the relative weakness of knee extensors in comparison with hamstrings in children with SMA [5, 22–24]. Although DMT treatment may postpone contracture development, our data indicate a similar vulnerability pattern as in studies performed before DMT were introduced [12, 14] suggesting that continued and protocolized surveillance is needed to identify children at risk. The fact that muscle weakness is more pronounced in legs than in arms in children with SMA might explain why elbow extension (despite the relatively severe weakness of triceps in comparison with biceps muscles [5]) and wrist range of motion in our cohort remained stable over time.

While the overall results show a decrease in knee extension range of motion of, on average, three degrees per year, the range of individual changes was large. This implies that some children may be more vulnerable than others and underlines the importance of surveillance of children with a routine check-up by a trained assessor every 6 months. Additional file 1 contains an example of a standardized protocol to this end. In our cohort, a decline in knee extension range of motion was observed after the age of 2 years. Dutch rehabilitation teams often started using standing frames after the age of 2 years, indicating a mismatch of current preventive interventions and clinical need. Despite limited evidence for interventions targeting joint mobility in neuromuscular disorders [36, 37], international Standards of Care [38] advocate early implementation of supported active and prolonged passive stretching [39]. Given that knee extension was the earliest and most affected joint motion, early preventive strategies—such as stretching, orthotic support, and/or standing frames—should be initiated probably in the first year of life and at least prior to detectable decrease in range of motion.

### Strengths and limitations

The strengths of the present study are the longitudinal observations across the age range of 0 to 8 years in a population-based sample. Range of motion measurements included knee and elbow extension and wrist deviation. Knee and elbow extension are of particular interest because of the characteristically severe weakness of the quadriceps and triceps muscles as compared to arm and leg flexors [5, 22–24]. Contractures of knee, elbow and wrist can greatly impact the level of functioning and participation. A child’s ability to stand and make transfers, to handle objects and to successfully and independently perform daily activities such as eating, combing hair and writing depend on a sufficient ROM of knee, elbow and wrist.

We acknowledge that our study also has limitations. Even though joint range of motion assessment by trained clinicians is a reliable method to determine the quantitative range of motion in neuromuscular disorders [40–42], performing goniometry in young children is challenging. We used parental assistance, timing of measurements shortly after feeding, and other commonly used approaches in clinical practice with young children to facilitate measurements. Our study started in the COVID19 period, during which rescheduling of outpatient appointments could not be avoided. This largely explains missing data.

Differences in prevalence of contractures could also be influenced by differences in contracture management strategies in children with SMA. Even though we collected data on the use of equipment and therapies for contracture management during the study, we did not collect such information in the period before children were enrolled. Moreover, we do not know the details of received contracture management, such as the duration and intensity while children were in follow-up in this longitudinal study. In addition, patient-reported outcomes on contracture management are susceptible to socially desirable responses. Nevertheless, for this study the patient-reported outcomes provided the most feasible means of capturing information on contracture management.

We did not include measurements of ankle range of motion (dorsiflexion/plantarflexion) or elbow pronation/supination, even though these ranges of motion are relevant for limb functionality. Consistent with Fujak et al., [12] contractures typically develop first at the knee, followed by the ankle and hip, which led us to prioritize the collection of knee data as a starting point in our study design. The reliability and validity of goniometric elbow measurements in adults show high intra- and interrater reliability for pro- and supination [43] Our clinical experience in young children, however, is that these measurements are highly challenging. Therefore, we prioritized wrist deviation measurements.

### Future studies

Many questions about contracture development remain unanswered and require further research. The origin of contractures is still under debate and appears to be multifactorial [39]. Contributing factors hypothesized are muscle weakness, leading to a decreased ability to actively move a limb through its full range of motion; static positioning for prolonged periods of time; and imbalance of agonist and antagonist muscles [14, 44]. Primary or secondary muscle changes such as fatty infiltration that may affect muscle length and flexibility [45, 46], that are unevenly distributed between agonists and antagonists could also contribute to contracture development [45]. Studies that aim at further dissecting the underlying mechanism of contracture development are needed, to explore why some joints are affected differently in treated SMA.

Our study provides a start for further research in children with SMA treated with DMT. Specific clinical characteristics that may determine the course of joint range of motion may include baseline motor function, type of DMT and age at start of DMT. Unfortunately, the sample size of our study was not sufficient to examine the independent contribution of these factors. We recommend that future studies examine the trajectory of joint range of motion in age groups beyond those investigated in the present study and include at a minimum knee/elbow extension and wrist deviation.

## Conclusion

This is the first longitudinal, population-based cohort study on contracture development in children with SMA, aged 0–8 years old, who receive DMT. Our study shows a high risk of loss of range of motion for the knee extension and a low risk of range of motion decline in elbow and wrist. This study demonstrates the importance of systematically assessing joint range of motion using a standardized protocol.

## Supplementary Information

Below is the link to the electronic supplementary material.

Supplementary Material 1: Additional file 1: Procedures manual. Standard operating procedure, which includes a detailed description of testing positions and goniometer placement based on standard goniometric measurements

Supplementary Material 2: Additional file 2: Contracture management questionnaire

Supplementary Material 3: Additional file 3: Range of motion data for subgroups with either 2 or 3 SMN2 copies

## Data Availability

The datasets used and/or analysed during the current study are available from the corresponding author on reasonable request.
