# Supplementary material for: Course of joint range of motion in children with spinal muscular atrophy receiving disease-modifying treatment
Source: Orphanet J Rare Dis. 2025 Nov 19;20:592. doi: 10.1186/s13023-025-04109-0 (PMC12628961; doi:10.1186/s13023-025-04109-0)
Supplement: Supplementary file 1 — Supplementary Material 1: Additional file 1: Procedures manual. Standard operating procedure, which includes a detailed description of testing positions and goniometer placement based on standard goniometric measurements [file 13023_2025_4109_MOESM1_ESM.docx]

**Appendix 1: Procedures Manual**

Assessment of passive range of motion of bilateral joint mobility of wrist, elbow and knee. Measurements will be taken with a universal plastic 18 cm goniometer.

*Reference ‘Handleiding: Standaard Lichamelijk Onderzoek’ (excluding wrist)^23^*

**Assessment to include passive joint range of motion of:**

1. **Wrist**: ulnar/radial deviation
2. **Elbow**: flexion/extension
3. **Knee**: flexion/extension and popliteal angle

Regarding the force applied by the examiner, we aim for the following:

1. The extreme angle of joint mobility will be determined by three gentle bouncing motions to the end range, followed by measurement of the maximum range of motion.

**Passive Range of Motion of the Wrist**

**Ulnar/Radial Deviation**

**Patient Position:**

- Sitting with the upper arm hanging down alongside the body, elbow in 90-degree flexion, and forearm in 90-degree pronation.
- If the patient cannot sit independently:
  - Sitting in a wheelchair with the upper arm hanging down as far as possible alongside the body or supported on an armrest, elbow in 90-degree flexion, and forearm in pronation.
- If measured with patient lying down in supine position:
  - Lying on the back, upper arm on the examination table, elbow in 90-degree flexion, and forearm in pronation.

**Examiner Position:**

- Standing in front of the patient.
- Forearm is supported on a table/bench/armrest/wheelchair tray with the elbow in 90-degree flexion.
- Executing hand at the level of the patient's hand.

**Execution:**

- Movement is performed separately for the left and right hand:
  - Move the hand into maximum radial deviation while keeping the elbow in the same position.
  - Move the hand into maximum ulnar deviation while keeping the elbow in the same position.
- Ensure the forearm does not change position.

**Joint Axis Location:**

- Parallel to the forearm.

**Goniometer Arms:**

- **Proximal:** Over the forearm (positioned centrally between the radius and ulna proximally).
- **Distal:** Over the hand at digit 3 (follows metacarpal 3).
- **Goniometer pivot:** Distally between the radius and ulna (proximal to the base of MC-3).

**Joint Position 0°:**

- Neutral wrist position (no palmar or dorsal flexion).

**PROM Score (degrees):**

- Measured angle = angle between digit 3 and forearm.
- Documented angle = angle between neutral position and ulnar/radial deviation.

**Passive Range of Motion of the Elbow**

**Flexion**

**Patient Position:**

- Sitting with the shoulder in 90-degree anteflexion, elbow in maximum extension, forearm as far as possible in 90-degree supination.
- If the patient cannot sit independently:
  - Sitting in a wheelchair, shoulder as far as possible in anteflexion, elbow/upper arm supported on a table/wheelchair tray/examination table.
- If measured lying down:
  - Lying on the back on the examination table, upper arm resting on the table, elbow in maximum extension, forearm in supination as far as possible.

**Examiner Position:**

- Standing beside or behind the patient.
- Supporting the upper arm on a table/wheelchair tray/examination table.
- Executing hand at the distal forearm of the patient.

**Execution:**

- Movement is performed separately for the left and right arm:
  - Bring the ipsilateral elbow into maximum flexion while keeping the executing hand at the distal forearm.
  - The supporting hand stabilizes the upper arm.

**Joint Axis Location:**

- Lateral epicondyle of the humerus.

**Goniometer Arms:**

- **Proximal:** Alongside the upper arm towards the humeral head.
- **Distal:** Alongside the forearm towards the radial head.

**Joint Position 0°:**

- Full elbow extension.

**PROM Score (degrees):**

- Measured angle = angle between upper arm and forearm.
- Documented angle = 180° - [angle between upper arm and forearm].


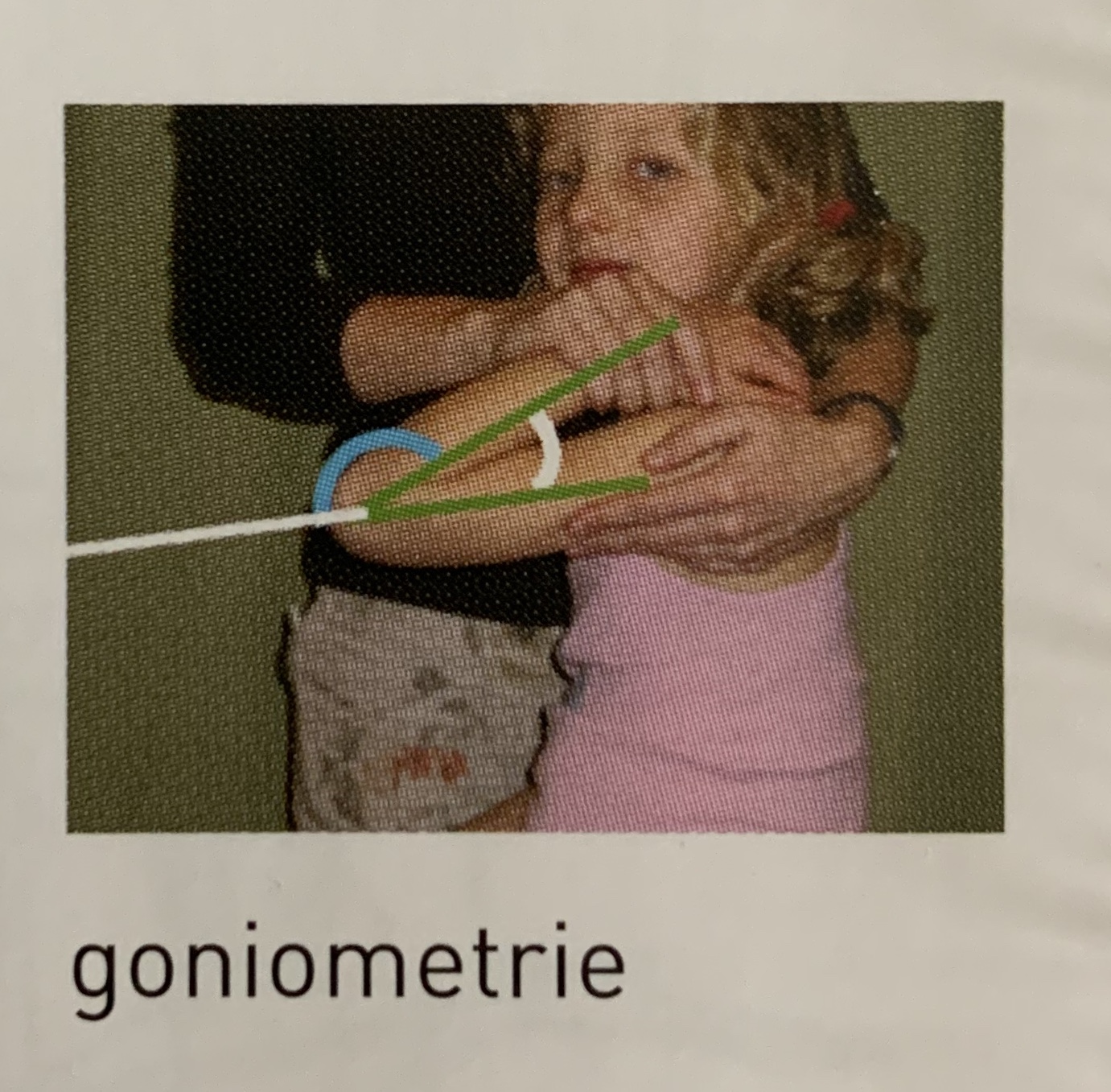
**Ref: ‘Standaard Lichamelijk onderzoek’^23^*

**Example:** The measured (white) angle is 25°; the documented (blue) angle is 180° - 25° = 155°.

**Passive Range of Motion of the Elbow**

**Extension**

**Patient Position:**

- Sitting with the shoulder in 90-degree anteflexion, elbow in maximum flexion, forearm in supination as far as possible.
- If measured lying down:
  - Lying on the back on the examination table, upper arm resting on the table, elbow in maximum flexion, forearm in supination as far as possible.
  - Note: If supination is incomplete, measure the angle in the sagittal plane.

**Examiner Position:**

- Standing beside or behind the patient.
- Supporting hand under the patient's upper arm or via examination table support.
- Executing hand at the distal forearm of the patient.

**Execution:**

- Movement is performed separately for the left and right arm:
  - Bring the ipsilateral elbow into maximum extension while keeping the executing hand at the distal forearm.
  - The supporting hand stabilizes the upper arm.

**Joint Axis Location:**

- Lateral epicondyle of the humerus.

**Goniometer Arms:**

- **Proximal:** Alongside the upper arm towards the humeral head.
- **Distal:** Alongside the forearm towards the radial head.

**Joint Position 0°:**

- Full elbow extension.

**PROM Score (degrees):**

- Measured angle = angle between upper arm and forearm.
- Documented angle = [angle between upper arm and forearm] – 180°.


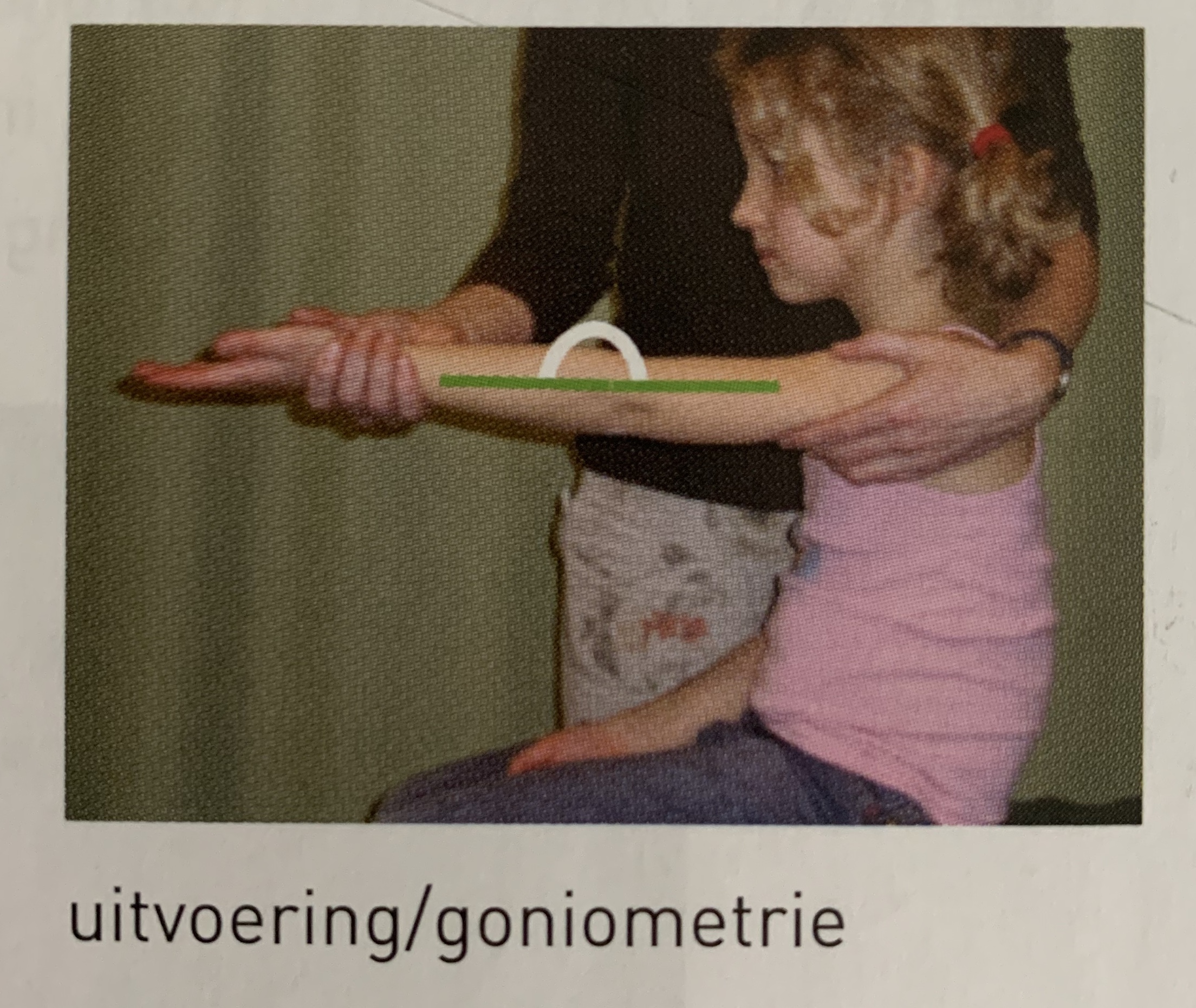
**Ref: ‘Standaard Lichamelijk onderzoek’^23^*

**Example:** The measured (white) angle is 180°; the documented angle is 180°-180° = 0°.

**Passive Range of Motion of the Knee**

**Flexion (Supine Position)**

**Patient Position:**

- Lying on the back with extended hips and knees.

**Examiner Position:**

- Standing beside the examination table at the patient’s pelvis level.
- Supporting hand on the patient’s knee.
- Executing hand at the patient’s ankle.

**Execution:**

- Movement is performed separately for the left and right leg:
  - Flex the ipsilateral knee maximally with the executing hand while the contralateral leg rests on the examination table. It does not matter if the contralateral leg also flexes.

**Joint Axis Location:**

- Lateral joint space at the lateral collateral ligament.

**Goniometer Arms:**

- **Proximal:** Along the lateral side of the thigh towards the greater trochanter.
- **Distal:** Along the lateral side of the lower leg, between the fibular head and the most lateral part of the malleolus.

**Joint Position 0°:**

- Full knee extension.

**PROM Score (degrees):**

- Measured angle = angle between upper and lower leg.
- Documented angle = 180° - [angle between upper and lower leg].


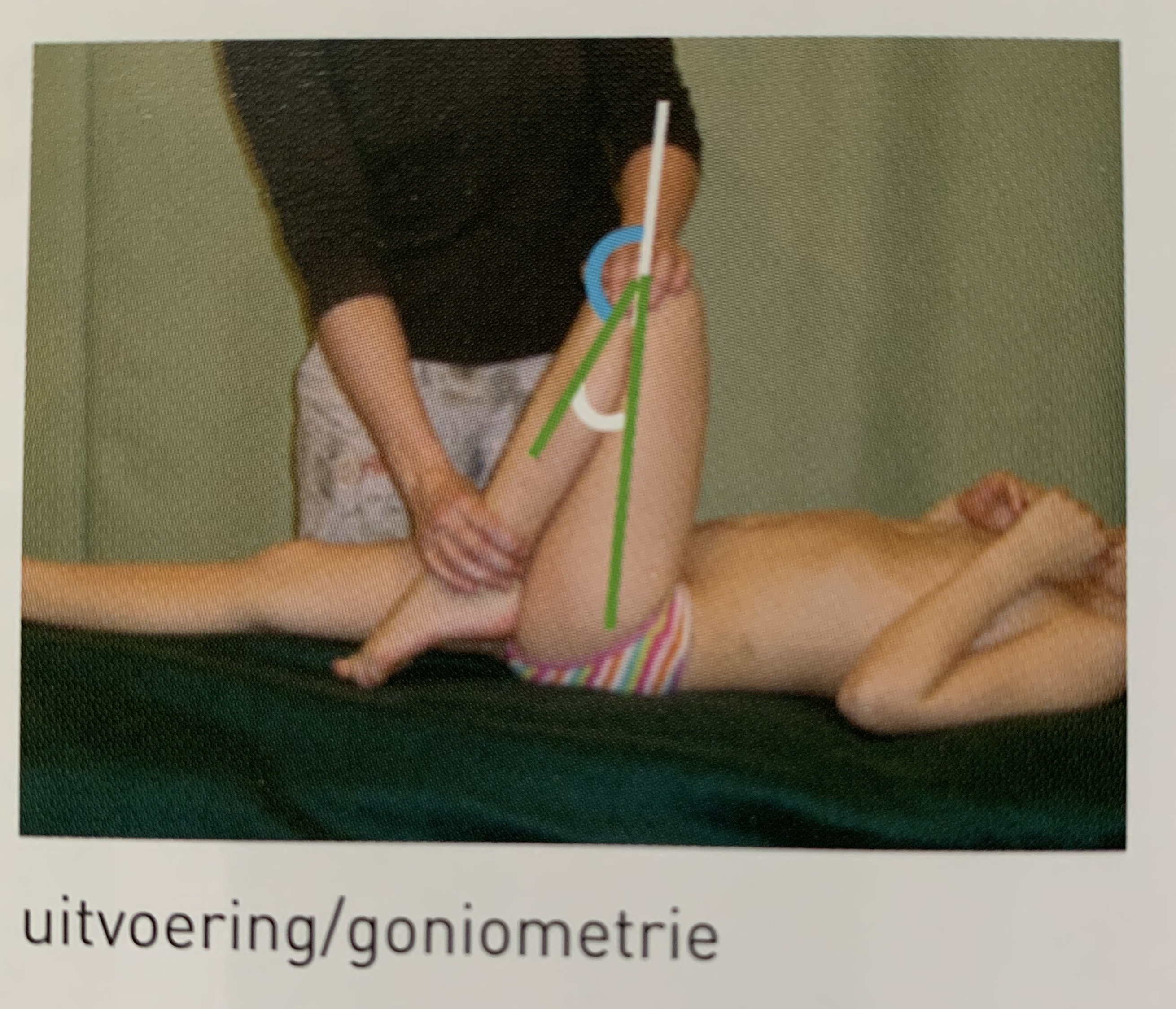


**Ref: ‘Standaard Lichamelijk onderzoek’^23^*

**Example:** The measured (white) angle is 30°; the documented (blue) angle is 180° - 30° = 150°.

**Passive Range of Motion of the Knee**

**Flexion (Prone Position)**

**Patient Position:**

- Lying prone with extended hips and knees.
- Regarding contractures: If the child’s navel touches the examination table, mobility is sufficient for inclusion, and knee flexion can be measured in the prone position.

**Examiner Position:**

- Standing next to the examination table at the patient’s pelvis level.
- Supporting hand placed on the patient’s pelvis.
- Executing hand positioned at the patient’s ankle.

**Execution:**

- Movement is performed separately for the left and right leg:
  - Flex the ipsilateral knee using the executing hand while the supporting hand stabilizes the pelvis. This hand monitors whether the pelvis lifts or rotates sideways.

**Joint Axis Location:**

- Lateral joint space at the lateral collateral ligament.

**Goniometer Arms:**

- **Proximal:** Along the lateral side of the thigh towards the greater trochanter.
- **Distal:** Along the lateral side of the lower leg, between the fibular head and the most lateral part of the malleolus.

**Joint Position 0°:**

- Full knee extension.

**PROM Score (degrees):**

- Measured angle = angle between upper and lower leg.
- Documented angle = 180° - [angle between upper and lower leg].


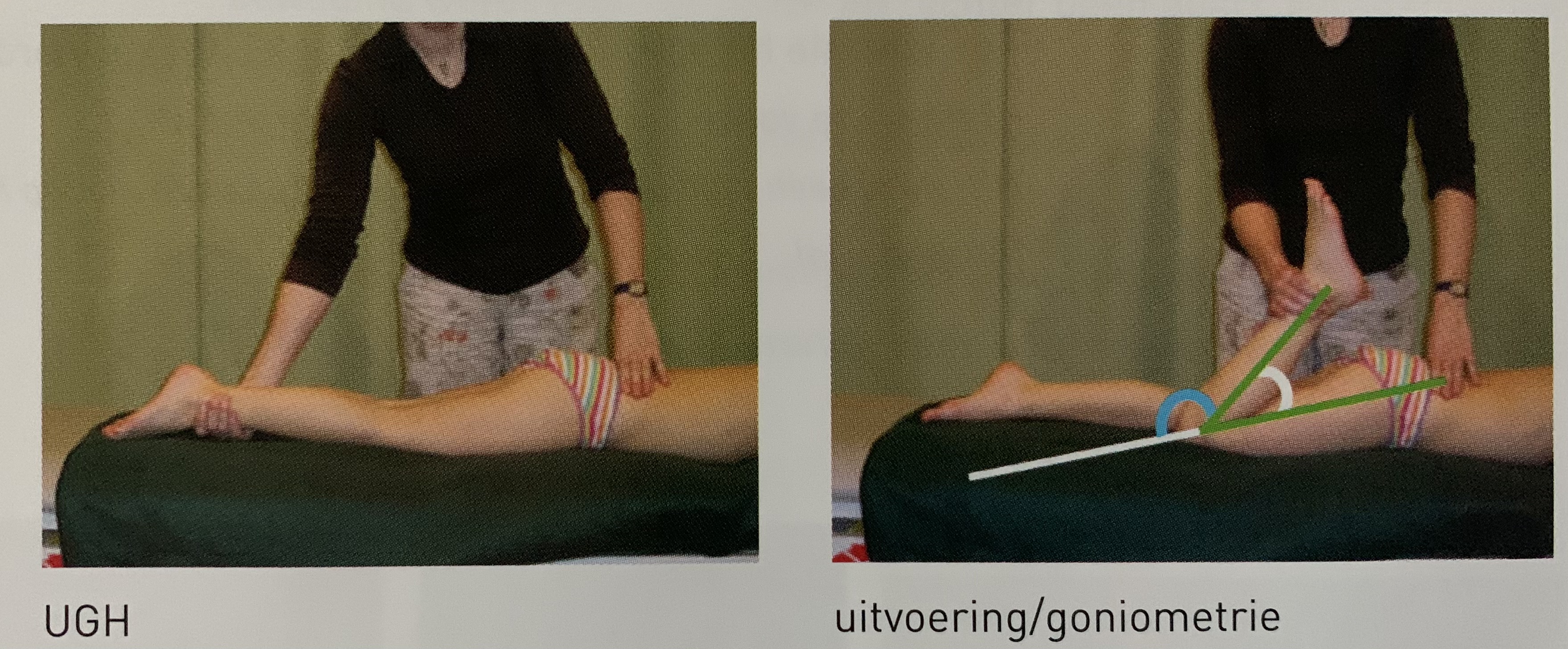


**Ref: ‘Standaard Lichamelijk onderzoek’^23^*

**Example:** The measured (white) angle is 40°; the documented (blue) angle is 180° - 40° = 140°.

**Note:** If the procedure is difficult due to a highly mobile child (e.g., young children who are difficult to instruct), parents may be asked to stabilize the child at the pelvic crest. The examiner performs the first measurement to ensure that the pelvic crest does not easily lift, confirming the accuracy of measurements when parents assist in stabilizing. This allows the examiner to use both hands freely for measurement.

**Passive Range of Motion of the Knee**

**Extension**

**Patient Position:**

- Lying on the back with extended hips and knees, heel resting on the table (contrary to the illustration where it is shown elevated).

**Examiner Position:**

- Standing beside the examination table at knee level.
- Supporting hand on the patient’s knee.

**Execution:**

- Movement is performed separately for the left and right leg:
  - Extend the ipsilateral knee using the executing hand while the contralateral leg remains resting on the examination table.
  - Force may be applied if necessary.

**Joint Axis Location:**

- Lateral joint space at the lateral collateral ligament.

**Goniometer Arms:**

- **Proximal:** Along the lateral side of the thigh towards the greater trochanter.
- **Distal:** Along the lateral side of the lower leg, between the fibular head and the most lateral part of the malleolus.

**Joint Position 0°:**

- Full knee extension.

**PROM Score (degrees):**

- Measured angle = angle between upper and lower leg.
- Documented angle = [angle between upper and lower leg] - 180°.


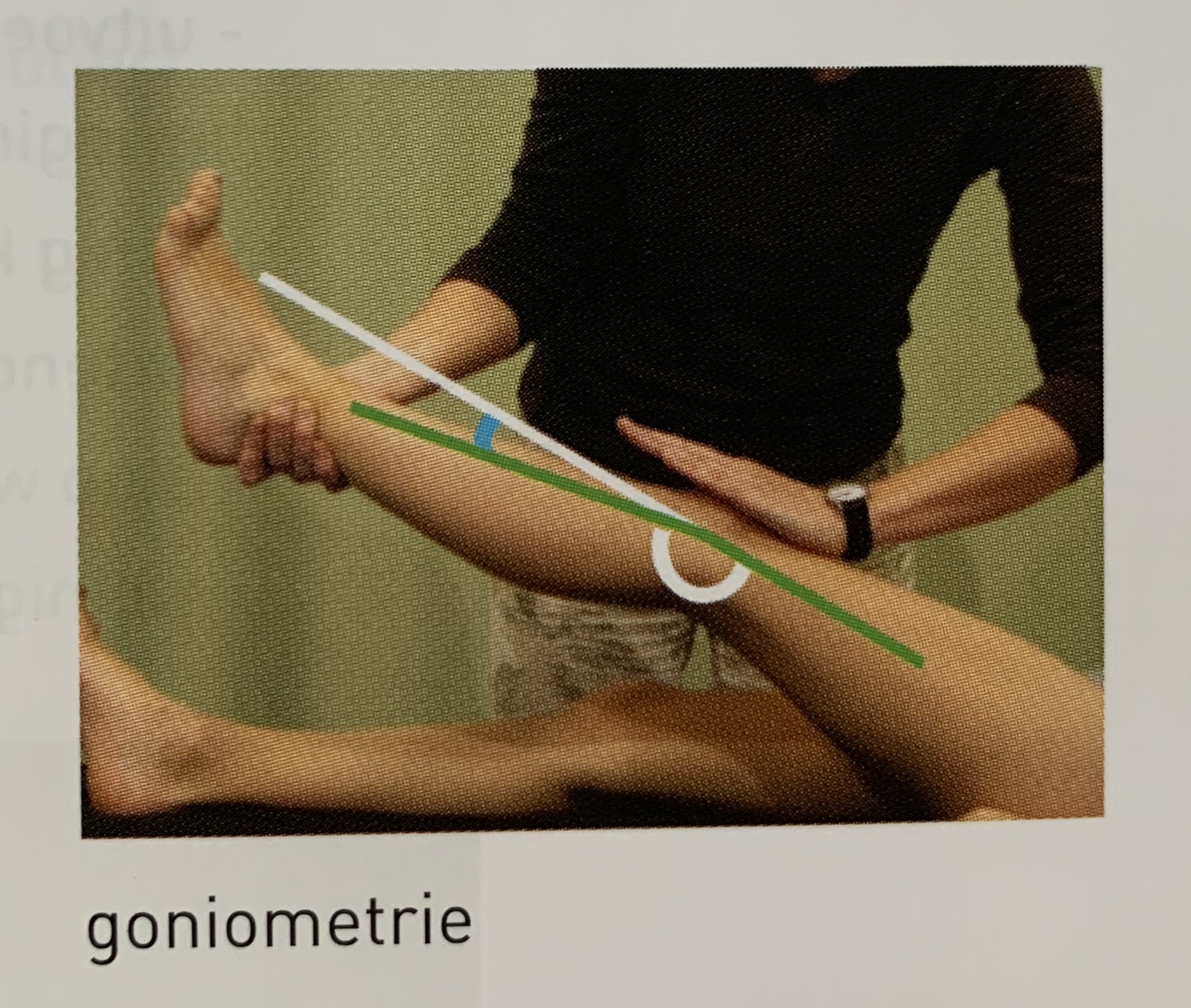
**Ref: ‘Standaard Lichamelijk onderzoek’^23^*

**Example:** The blue angle indicates a limitation in left knee extension. The measured (white) angle is 170, the documented (blue) angle is 170° - 180° = -10°.

**Passive Range of Motion of the Knee**

**Extension in Hip Flexion (Popliteal Angle)**

**Patient Position:**

- Lying on the back with 90-degree hip flexion and maximally flexed knee on the ipsilateral leg, while the contralateral leg rests on the examination table.
- Note: If knee flexion contracture is present in the contralateral leg, position the patient at the edge or diagonally on the examination table, allowing the lower leg to hang over the edge (keeping the hip in extension). Not feasible for some children with SMA, due to anxiety and reduced muscle strength.

**Examiner Position:**

- Standing beside the examination table at pelvis level.
- Supporting hand on the patient’s knee.
- Executing hand at the patient’s ankle.

**Execution:**

- Movement is performed separately for the left and right leg, using a gentle bouncing motion with minimal force:
  - Extend the ipsilateral knee using the executing hand.
  - Stop when the contralateral leg starts to move.
  - Apply minimal force.

**Joint Axis Location:**

- Lateral joint space at the lateral collateral ligament.

**Goniometer Arms:**

- **Proximal:** Along the lateral side of the thigh towards the greater trochanter.
- **Distal:** Along the lateral side of the lower leg, between the fibular head and the most lateral part of the malleolus.

**Joint Position 0°:**

- Full knee extension.

**PROM Score (degrees):**

- Measured angle = angle between upper and lower leg.
- Documented angle = 180° - [angle between upper and lower leg].


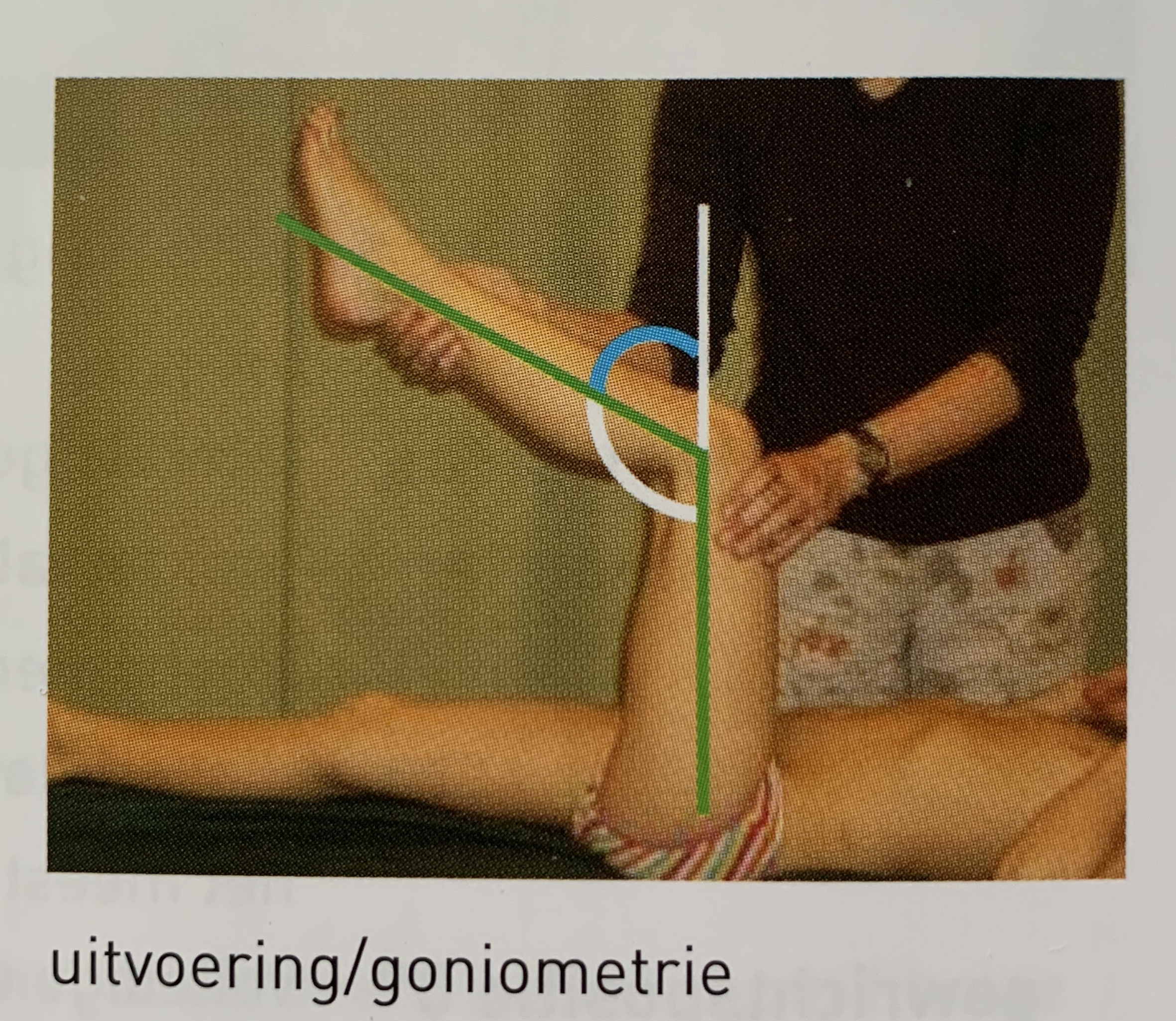
**Ref: ‘Standaard Lichamelijk onderzoek’^23^*

**Example:** The measured (white) angle is 115°; the documented (blue) angle is 180° - 115° = 65°.
