# Supplementary material for: Course of joint range of motion in children with spinal muscular atrophy receiving disease-modifying treatment
Source: Orphanet J Rare Dis. 2025 Nov 19;20:592. doi: 10.1186/s13023-025-04109-0 (PMC12628961; doi:10.1186/s13023-025-04109-0)
Supplement: Supplementary file 2 — Supplementary Material 2: Additional file 2: Contracture management questionnaire [file 13023_2025_4109_MOESM2_ESM.docx]

**Contracture management questionnaire**

*The following questions concern the preventive treatment of joint deformities (contractures) in your child. When completing the questionnaire, we ask you to think of an average week in the recent period.*

Stretching:

- Does your child receive physiotherapy? Yes/No
- **If 'Does your child receive physiotherapy?' is equal to 'yes' answer this question:**

Are stretching exercises for the joints performed during this therapy? Yes/No

- Do you perform stretching exercises with your child yourself? Yes/No
- Are there other healthcare providers who mobilize your child’s joints? Yes/No

Orthosis/splints

- Does your child use orthosis? Yes/No
- **If ‘Does your child use orthosis’ is equal to ‘Yes’ answer this question:**

For which body parts does your child use orthosis? (multiple choice, multiple answers possible)

Feet? Knees? Wrists? Other orthosis?

Standing frame

- Does your child use a standing frame? Yes/No

*Thank you very much for completing this questionnaire. We look forward to seeing you at the hospital for the joint measurements.*
