# Supplementary figures and images for: Course of joint range of motion in children with spinal muscular atrophy receiving disease-modifying treatment
Source: Orphanet J Rare Dis. 2025 Nov 19;20:592. doi: 10.1186/s13023-025-04109-0 (PMC12628961; doi:10.1186/s13023-025-04109-0)

Joint Range of Motion – 2 SMN2 copies


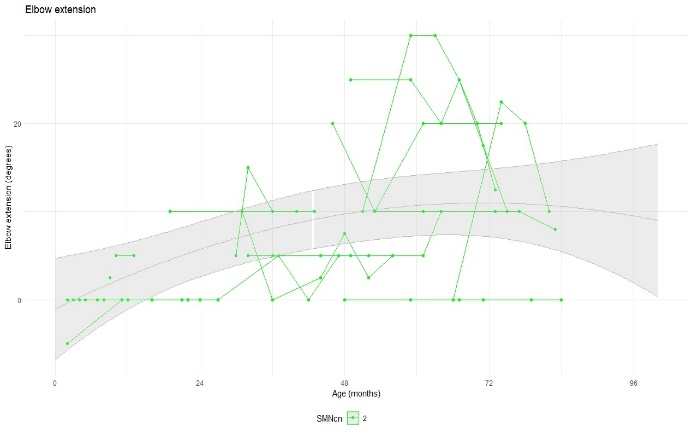

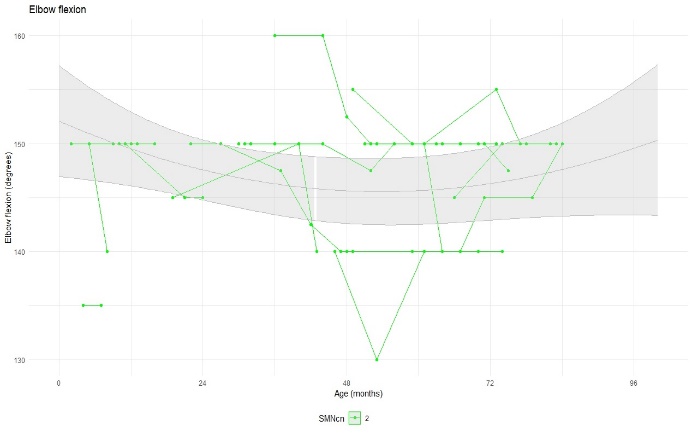

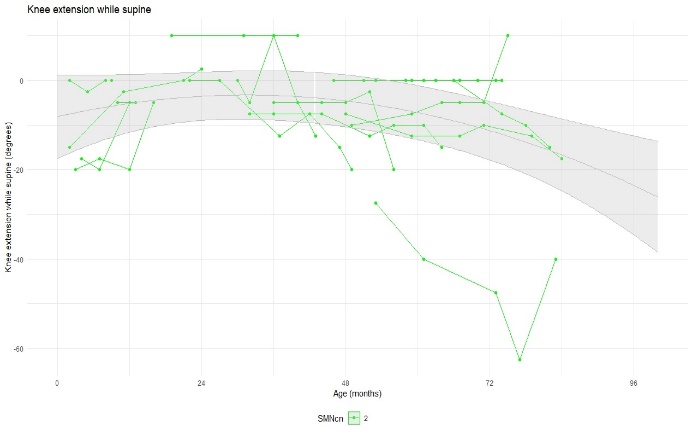

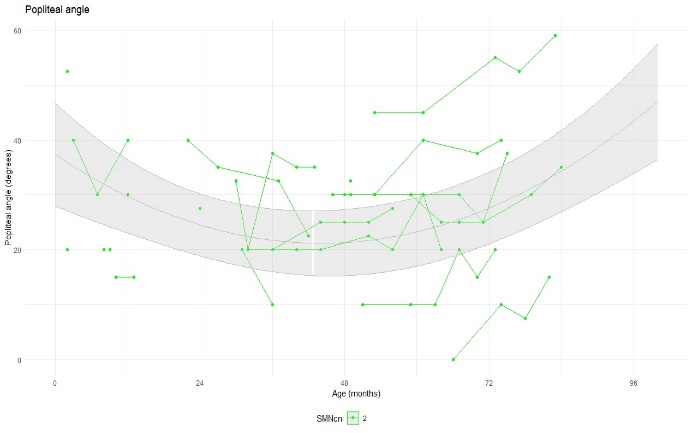

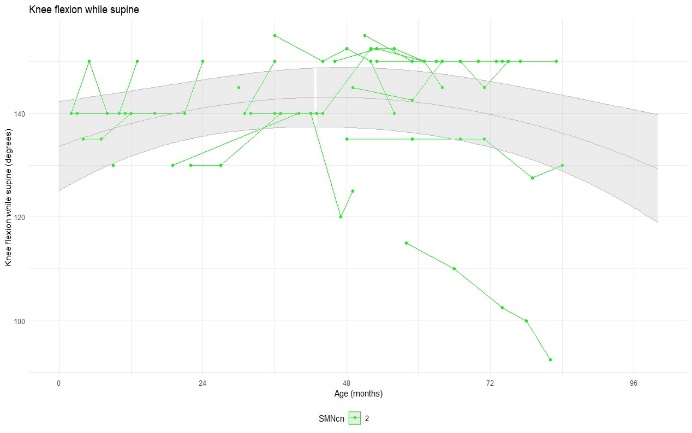

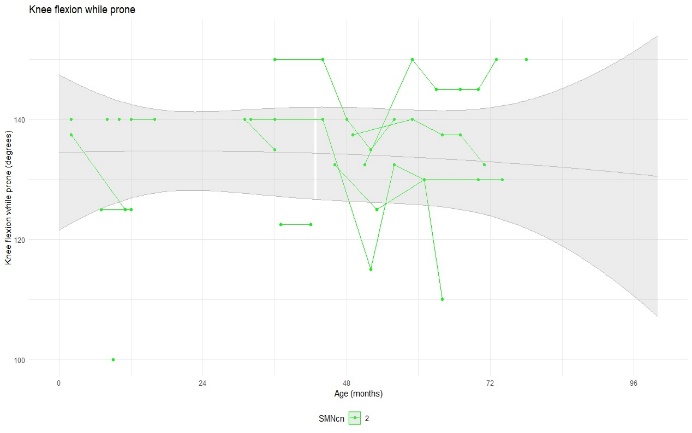

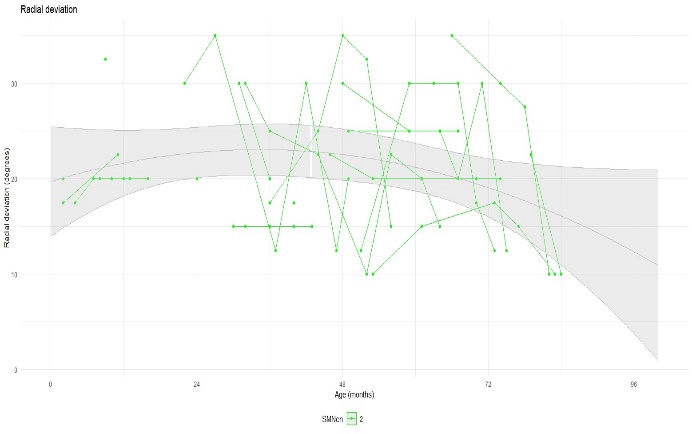

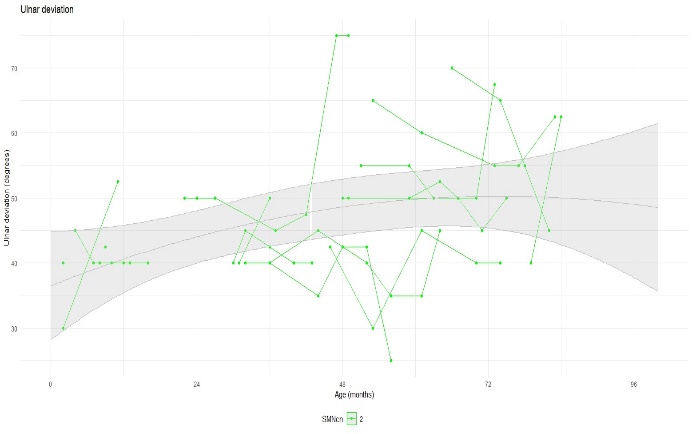


Joint Range of Motion – 3 SMN2 copies


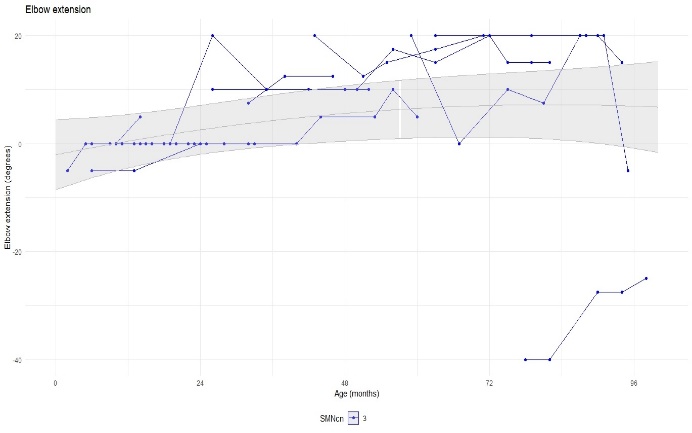

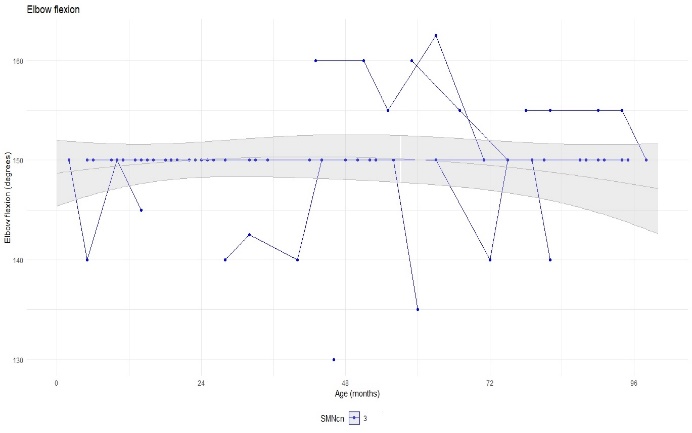

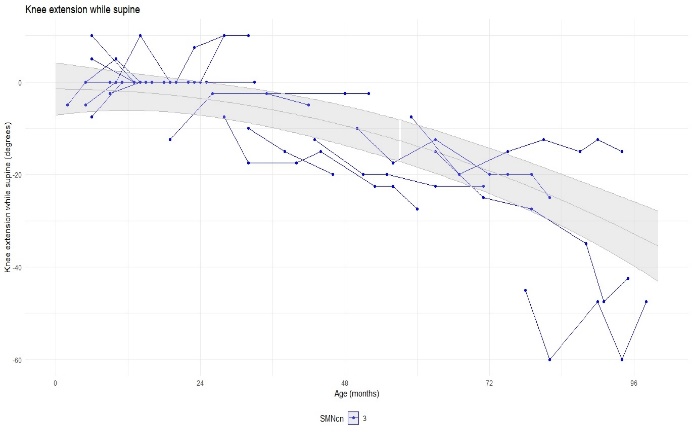

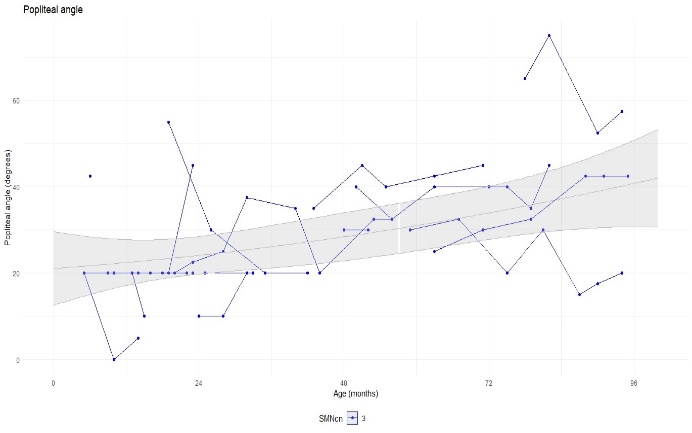

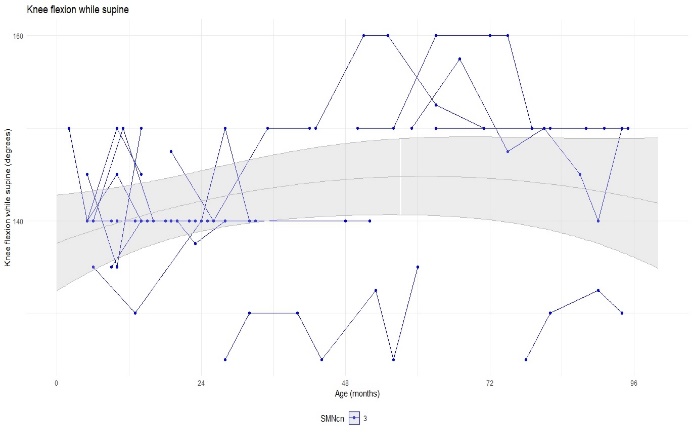

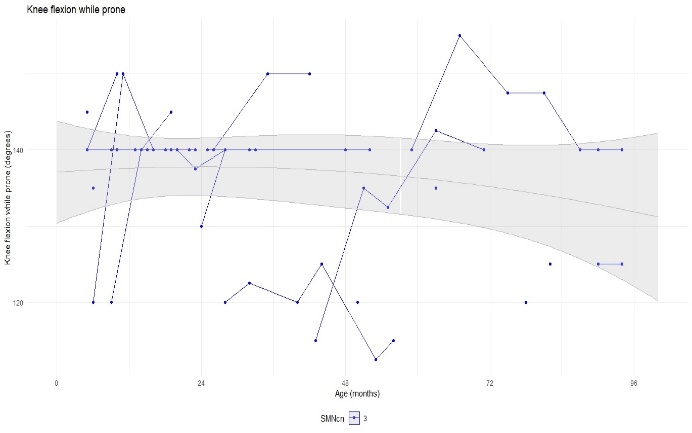


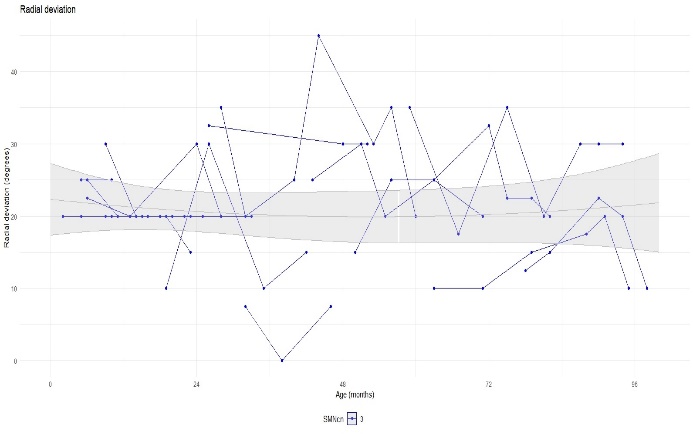

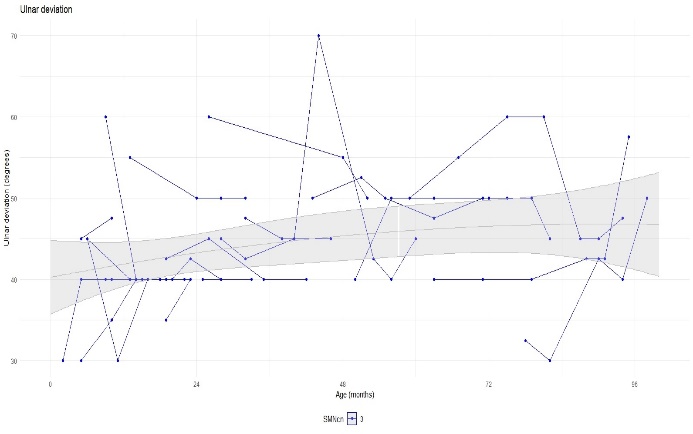

Supplement: Supplementary file 3 — Supplementary Material 3: Additional file 3: Range of motion data for subgroups with either 2 or 3 SMN2 copies [file 13023_2025_4109_MOESM3_ESM.docx]
